# The EULAR-OMERACT joint-level scoring of ultrasound synovitis demonstrates good construct validity when tested at the patient-level in comparison with measures of disease activity and joint damage in patients with rheumatoid arthritis

**DOI:** 10.3389/fmed.2025.1564381

**Published:** 2025-05-15

**Authors:** York Kiat Tan, Julian Thumboo

**Affiliations:** ^1^Department of Rheumatology and Immunology, Singapore General Hospital, Singapore, Singapore; ^2^Duke-NUS Medical School, Singapore, Singapore; ^3^Yong Loo Lin School of Medicine, National University of Singapore, Singapore, Singapore

**Keywords:** synovitis, rheumatoid arthritis, ultrasound, joints, bone erosion

## Abstract

**Objective:**

Patient-level ultrasound joint inflammation outcomes, derived from the European Alliance of Associations for Rheumatology-Outcome Measures in Rheumatology (EULAR-OMERACT) joint-level scoring of elementary components and the combined score (CS), were compared with measures of disease activity and joint damage in patients with rheumatoid arthritis (RA).

**Methods:**

Clinical joint assessment and a 22-joint (bilateral hands/wrists) ultrasonography were performed independently during the same patient study visit. Patient-level ultrasound joint inflammation outcomes [total power Doppler (PD) score, total grayscale (GS) score, total CS, number of joint(s) with CS ≥ 2 (at least moderate synovitis), and number of joint(s) with ultrasound synovitis defined as PD > 0 or GS ≥ 2] derived from the EULAR-OMERACT joint-level scoring system were correlated with Clinical Disease Activity Index (CDAI), 28-joint disease activity score (DAS28), and ultrasound-detected joint damage, i.e., total bone erosion score (TBES). The relationship between the variables was studied using simple linear regression.

**Results:**

A total of 83 RA patients underwent scanning of 1,826 joints in this cross-sectional study. All patient-level ultrasound joint inflammation outcomes showed significant correlations (*p* < 0.01) with CDAI, DAS28, and TBES (with correlation coefficients ranging from 0.45 to 0.48, 0.38 to 0.45, and 0.66 to 0.83, respectively). A linear regression analysis revealed statistically significant relationships (*p* < 0.01) for all patient-level ultrasound joint inflammation outcomes in relation to CDAI, DAS28, and TBES (with regression coefficients ranging from 0.603 to 1.260, 0.066 to 0.149, and 0.416 to 0.818, respectively).

**Conclusion:**

Patient-level ultrasound joint inflammation outcomes, derived from the EULAR-OMERACT joint-level scoring system, showed good construct validity when compared to both disease activity and joint damage in patients with RA.

## 1 Introduction

Over the past two decades, alongside growing popularity among rheumatologists worldwide, there has been a substantial amount of knowledge gained in the use of musculoskeletal ultrasound imaging for joint assessment in patients with rheumatoid arthritis (RA) (1). In the early 2000s, the Outcome Measures in Rheumatology (OMERACT) ultrasound special interest group, recognizing the great potential of ultrasound as an outcome measure in RA trial settings [including its good patient tolerability and feasibility in scanning multiple joints over a short period of time when compared to magnetic resonance imaging (MRI) (2, 3)] has provided consensus definitions for common joint pathologies (such as synovial hypertrophy and bone erosion) in patients with inflammatory arthritis (3). Ultrasound offers the advantage of directly visualizing joint pathologies in RA, with features of joint inflammation (e.g., power Doppler (PD) synovial vascularity and synovial hypertrophy) being quantifiable and scorable based on their severity (4). Different grading systems (including semi-quantitative scoring of ultrasound synovitis, e.g., using a 0–3 severity scale) and joint counts for ultrasonography have been proposed for joint inflammation assessment in patients with RA (5). The advent of standardized definitions of ultrasound joint pathologies and the development of semi-quantitative scoring of joint inflammation have helped facilitate the growth of ultrasound as an outcome for synovitis assessment in both the RA trial and clinical settings (6, 7). Nonetheless, there remained no consensus on an ultrasound scoring system for RA clinical trials until the latter half of the 2010s, when the European Alliance of Associations for Rheumatology-OMERACT (EULAR-OMERACT) ultrasound task force developed a highly reliable, standardized, international, consensus-based RA ultrasound synovitis scoring system (8, 9). The validity of outcome measurement instruments can be assessed by applying the OMERACT filter, which consists of three main components: truth, discrimination, and feasibility (10, 11). Reliability testing is part of the discrimination component of the OMERACT filter (10, 11), and establishing a reliable consensus-based ultrasound scoring system for synovitis assessment has been an important contribution of the EULAR-OMERACT ultrasound task force in the development of ultrasound as an outcome measurement tool for joint inflammation assessment in patients with RA. This joint-level ultrasound synovitis scoring system (8, 9) was designed to evaluate the elementary components [i.e., grayscale (GS) and PD] using semi-quantitative scoring (0 to 3), along with a combined score (CS). The latter (CS) is also scored semi-quantitatively (0 to 3), with its severity grading derived from combinations of the severity scores of the elementary components (8, 9). Apart from inter- and intra-observer reliability, it is important to test the EULAR-OMERACT ultrasound synovitis scoring system (8, 9) according to other aspects of the OMERACT filter—e.g., construct validity (as part of the truth component of the OMERACT filter), sensitivity to change (as part of the discrimination component of the OMERACT filter), feasibility for use, etc. (10, 11)—and to determine its usefulness at the patient level. Relatively limited additional validation studies in patients with RA have been conducted since the development of the highly reliable EULAR-OMERACT ultrasound synovitis scoring system (8, 9). One retrospective study demonstrated patient-level correlations between the summed GS and PD scores (without evaluating the CS) at the hands/wrists and RA disease activity (12), while another small-scale study (13) compared GS, PD, and combined scores with clinical, imaging [magnetic resonance imaging (MRI) and X-ray], and histology variables at the RA wrist. Currently, there are no well-established patient-level ultrasound joint inflammation outcomes (s) derived from the EULAR-OMERACT joint-level ultrasound synovitis scoring system recommended for use in patients with RA; hence, further investigative efforts in this area may be necessary. This present study aims to test the construct validity of patient-level ultrasound joint inflammation outcomes derived from the EULAR-OMERACT joint-level ultrasound synovitis scoring system (8, 9) by comparing them with measures of disease activity and joint damage in patients with RA.

## 2 Materials and methods

### 2.1 Study population

This single-center, cross-sectional study was conducted at the Singapore General Hospital, a tertiary referral center and flagship hospital of the SingHealth Duke NUS Academic Medical Centre. The inclusion criteria were as follows: (1) male or female participants aged from 21 to 99 years, (2) meeting the 2010 EULAR/American College of Rheumatology (ACR) classification criteria for RA (14), (3) disease duration of <2 years, and (4) treatment with first-line conventional disease-modifying anti-rheumatic drugs (DMARDs). Pregnant women were excluded from the study. Patients who were eligible for the study were consecutively recruited at the rheumatology outpatient clinic of the hospital from December 2020 to January 2024. Our study, which was approved by the SingHealth Centralised Institutional Review Board (CIRB) (2020/2669), adheres to the relevant ethical guidelines for research. All patients provided their informed consent prior to recruitment into the study.

### 2.2 Clinical assessment

Clinical and ultrasound imaging assessments were performed during the same study visit. Trained rheumatology nurses (who received prior standardized training), who were blinded to the results from ultrasound imaging, performed the clinical joint assessment and scored the Clinical Disease Activity Index (CDAI) and the 28-joint disease activity score (DAS28) [both of which are measures of RA disease activity used in routine clinical practice (15)].

### 2.3 Imaging assessment

Ultrasound imaging was performed by a rheumatologist with more than 10 years of experience in musculoskeletal ultrasonography who was blinded to the findings of the clinical joint assessors. A Mindray M9 ultrasound machine (with machine settings of Doppler frequency, 5.7 MHz, and pulse repetition frequency, 700 Hz), along with an L14-6Ns linear probe, was utilized for the study. Ultrasonography was performed according to the EULAR recommendations (16) at the dorsal recesses of 22 joint sites as follows: wrists, metacarpophalangeal joints (MCPJs) 1 to 5, thumb interphalangeal joints, and proximal interphalangeal joints (PIPJs) 2 to 5 (i.e., index finger through to the little finger), bilaterally. For the wrist, the radiocarpal and midcarpal joints were assessed as a single site. The ultrasound elementary components (PD and GS synovial hypertrophy) and the CS were each graded semi-quantitatively on a 0 to 3 severity scale (i.e., 0 = normal, 1 = mild, 2 = moderate, and 3 = severe) based on the EULAR-OMERACT scoring system (8, 9). The semi-quantitative (0 to 3) grading of the EULAR-OMERACT CS (8, 9) is derived from combinations of the severity scores of the elementary components (GS and PD) and can be briefly summarized as follows: Grade 0 (normal): GS = 0 with PD = 0; Grade 1 (minimal): GS = 1 with PD ≤ 1; Grade 2 (moderate): GS = 2 with PD ≤ 2 or GS = 1 with PD = 2; and Grade 3 (severe): GS = 3 with PD ≤ 3 or GS = 1 or 2 with PD = 3. Ultrasound bone erosion score (BES) was graded as either 1 = present or 0 = absent at each joint based on the OMERACT definition of bone erosion (3). This dichotomous grading of bone erosion at each joint site means that, if no erosion is detected, the joint is assigned a score of “0,” whereas if one or more erosions are detected, the joint is assigned a score of “1.” The OMERACT definition of bone erosion (3) was adopted in the present study, as there is currently no consensus on grading bone erosion using more sophisticated scoring methods (such as semi-quantitative scoring).

### 2.4 Statistical analysis

For each patient, the PD score, GS score, and CS at the 22 joint sites were summed up to obtain the respective total PD score (TPDS), total GS score (TGSS), total CS (TCS), and total BES (TBES).

Patient-level ultrasound joint inflammation outcomes—TPDS, TGSS, TCS, number of joints with CS ≥ 2 [indicating at least moderate synovitis (8, 9)], and number of joints with ultrasound synovitis [PD > 0 or GS ≥ 2, as defined in previous studies (17, 18)]—were correlated with disease activity measures (i.e., CDAI and DAS28) and ultrasound-detected joint damage (i.e., TBES) using Spearman’s correlation coefficient. Their relationships were also studied using simple linear regression (which estimates the relationship between a dependent variable and an independent variable). Statistical analyses were performed using SPSS version 26.

## 3 Results

### 3.1 Baseline patient characteristics

A total of 83 RA patients had 1,826 joints scanned in this cross-sectional study. The baseline characteristics of the patient cohort are as follows: mean (SD) age of 57.0 (12.4) years; *n* = 63 (76.0%) Chinese; *n* = 62 (74.7%) female; mean (SD) disease duration, DAS28, and CDAI: 6.4 (5.9) months, 3.7 (1.3), and 11.5 (11.4), respectively. All patients were on one or more of the following conventional DMARDs: methotrexate, leflunomide, sulfasalazine, and hydroxychloroquine; 56 out of the 83 patients (67.5%) were on oral prednisolone. The mean (SD) TPDS, TGSS, and TCS were 7.7 (6.8), 32.0 (8.6), and 31.4 (8.4), respectively. The median (IQR) of TPDS, TGSS, and TCS were 6 (8), 32 (13), and 30 (12), respectively.

### 3.2 Correlation analysis

All patient-level ultrasound joint inflammation outcomes (TPDS, TGSS, TCS, number of joint(s) with CS ≥ 2, and number of joint(s) with ultrasound synovitis showing PD > 0 or GS ≥ 2) correlated significantly (all *p* < 0.01) with disease activity measures (CDAI and DAS28) and ultrasound-detected joint damage (TBES). The strength of the correlation of the patient-level ultrasound joint inflammation outcomes appeared to be similar for both disease activity measures (CDAI: correlation coefficient ranging from 0.45 to 0.48 and DAS28: correlation coefficient ranging from 0.38 to 0.45), while the strength of their correlation appeared to be stronger with ultrasound-detected joint damage (TBES: correlation coefficient ranging from 0.66 to 0.83) (see Table 1).

**Table 1:** Correlation of patient-level ultrasound joint inflammation outcomes with disease activity and joint damage.

Patient-level ultrasound joint inflammation outcomes	Disease activity				Joint damage	
	CDAI		DAS28		Ultrasound TBES	
	Correlation coefficient	*p*-value	Correlation coefficient	*p*-value	Correlation coefficient	*p-*value
Total PD score	0.48	<0.001***	0.41	<0.001***	0.66	<0.001***
Total GS score	0.45	<0.001***	0.38	0.001**	0.82	<0.001***
Total CS	0.48	<0.001***	0.40	<0.001***	0.81	<0.001***
Number of joint(s) with CS ≥ 2	0.48	<0.001***	0.45	<0.001***	0.83	<0.001***
Number of joint(s) with PD > 0 or GS ≥ 2	0.45	<0.001***	0.40	0.002**	0.83	<0.001***

CDAI, Clinical Disease Activity Index; DAS28, 28-joint disease activity score; TBES, total bone erosion score; PD, power Doppler; GS, grayscale; CS, combined score. Statistical significance: *p* < 0.01**, *p* < 0.001***.

### 3.3 Linear regression analysis

A linear regression analysis (see Table 2) revealed a statistically significant relationship (all *p* < 0.001) for all patient-level ultrasound joint inflammation outcomes (TPDS, TGSS, TCS, number of joint(s) with CS ≥ 2, and number of joint(s) with ultrasound synovitis showing PD > 0 or GS ≥ 2) versus disease activity measures [CDAI: for each independent variable (i.e., TPDS, TGSS, TCS, number of joint(s) with CS ≥ 2, and number of joint(s) with ultrasound synovitis showing PD > 0 or GS ≥ 2), a one-unit increase will increase the dependent variable (CDAI) by 0.922, 0.585, 0.603, 1.260, and 0.955, respectively; DAS28: for each independent variable (i.e., TPDS, TGSS, TCS, number of joint(s) with CS ≥ 2, and number of joint(s) with ultrasound synovitis showing PD > 0 or GS ≥ 2), a one-unit increase will increase the dependent variable (DAS28) by 0.099, 0.066, 0.069, 0.149, and 0.112, respectively;] and ultrasound-detected joint damage [TBES: for each independent variable (i.e., TPDS, TGSS, TCS, number of joint(s) with CS ≥ 2, and number of joint(s) with ultrasound synovitis showing PD > 0 or GS ≥ 2), a one-unit increase will increase the dependent variable (TBES) by 0.452, 0.416, 0.418, 0.818, and 0.683, respectively].

**Table 2:** Linear regression analysis of patient-level ultrasound joint inflammation outcomes versus disease activity and joint damage.

Patient-level ultrasound joint inflammation outcomes	Disease activity				Joint damage	
	CDAI		DAS28		Ultrasound TBES	
	β Coefficient (95% CI)	*p*-value	β Coefficient (95% CI)	*p-*value	β Coefficient (95% CI)	*p*-value
Total PD score	0.922 (0.609, 1.236)	<0.001***	0.099 (0.061, 0.137)	<0.001***	0.452 (0.351, 0.552)	<0.001***
Total GS score	0.585 (0.321, 0.849)	<0.001***	0.066 (0.035, 0.098)	<0.001***	0.416 (0.353, 0.479)	<0.001***
Total CS	0.603 (0.335, 0.872)	<0.001***	0.069 (0.038, 0.101)	<0.001***	0.418 (0.352, 0.484)	<0.001***
Number of joint(s) with CS ≥ 2	1.260 (0.753, 1.766)	<0.001***	0.149 (0.090, 0.209)	<0.001***	0.818 (0.694, 0.942)	<0.001***
Number of joint(s) with PD > 0 or GS ≥ 2	0.955 (0.527, 1.382)	<0.001***	0.112 (0.061, 0.162)	<0.001***	0.683 (0.584, 0.782)	<0.001***

CDAI, Clinical Disease Activity Index; DAS28, 28-joint disease activity score; TBES, total bone erosion score; PD, power Doppler; GS, grayscale; CS, combined score. Statistical significance: *p* < 0.001***.

## 4 Discussion

In our study, we evaluated the use of patient-level ultrasound joint inflammation outcomes derived from the joint-level EULAR-OMERACT scoring system (8, 9). To the best of our knowledge, our study is the first to demonstrate that the patient-level ultrasound joint inflammation outcomes derived from the joint-level EULAR-OMERACT scoring system have a significant association with both disease activity (CDAI and DAS28) and ultrasound-detected joint damage (TBES) in patients with RA. This is particularly pertinent since there is currently no consensus on which patient-level ultrasound joint inflammation outcome(s) derived from the EULAR-OMERACT joint-level ultrasound synovitis scoring system to use for RA joint inflammation assessment and disease monitoring. Through our study, we have proposed a set of patient-level ultrasound joint inflammation outcomes derived from the joint-level EULAR-OMERACT scoring system by applying ultrasonography to the bilateral hands/wrists of patients with RA. These outcomes, however, need to be further validated in other independent RA cohorts. In a retrospective study (*n* = 163 RA patients) by Zhao et al. (12) involving ultrasound of the hands/wrists, in addition to demonstrating high intra- and inter-observer reliability (Cohen’s kappa coefficient ranging from 0.72 to 0.97), a significant correlation (correlation coefficient ranging from 0.57 to 0.79) was observed between the sum of the GS and PD scores versus the DAS28-C-reactive protein (DAS28-CRP) (after the study investigators (radiologists) received standardized training on the EULAR-OMERACT scoring system). In a separate small-scale wrist study by Just et al. (13), in both the early RA (*n* = 20) and long-standing RA (*n* = 20) patient groups, the baseline EULAR-OMERACT ultrasound PD, GS, and CS all showed significant correlations with the RA MRI score (RAMRIS) MRI synovitis and synovial biopsy inflammation (as determined by the Krenn score) but not with the DAS28-CRP. At baseline, in the long-standing RA patient group, only the EULAR-OMERACT ultrasound CS was significantly correlated with the Larsen radiographic score, whereas only the EULAR-OMERACT PD and combined scores were significantly correlated with the MRI erosion score. The relatively small sample size of the study by Just et al. (13) may help explain the lack of significant correlation in some of the baseline comparative analyses. Moreover, ultrasound outcomes at the single joint level (i.e., wrist) may not necessarily be reflective of patient-level global RA disease activity (e.g., DAS28-CRP). Finally, conventional radiography (CR) is less sensitive than ultrasound in detecting bone erosions, especially in early RA (19), which may explain why none of the baseline EULAR-OMERACT ultrasound scores showed a significant correlation with the Larsen X-ray score in the early RA patient group (18). Future well-designed imaging studies with larger sample sizes will be necessary to allow a more robust analysis in patients with RA.

Patient-level ultrasound joint inflammation outcomes in our study were found to be more strongly correlated with ultrasound-detected joint damage (TBES: correlation coefficient ranging from 0.66 to 0.83) when compared to measures of disease activity (CDAI and DAS28: correlation coefficient ranging from 0.38 to 0.48). In our study, ultrasonography was performed at 22 joint sites, whereas in the calculation of CDAI and DAS28, data were derived from swollen and tender joint counts at 28 joint sites, which may explain the difference in the strength of the correlation. Although more time-consuming, the correlation between patient-level ultrasound joint inflammation outcomes and disease activity may increase with ultrasonography at more joint sites. An important research agenda for future ultrasound studies incorporating and validating the EULAR-OMERACT scoring system for use in RA clinical trials would be to determine whether any optimal reduced ultrasound joint set(s) or combination(s) exist. Scanning a reduced joint set would be less time-consuming, thereby increasing its feasibility compared to scanning an extended number of joint sites (5, 20).

Our study has several limitations. The clinical and ultrasound assessments were performed at a single time-point using a cross-sectional study design among early RA patients (disease duration <2 years) on conventional DMARDs. A recent small-scale (*n* = 52) prospective study (21) by Germanò et al. utilized the EULAR-OMERACT ultrasound synovitis scoring system and demonstrated clinical improvement (e.g., DAS28) along with a reduction in ultrasound-detected joint inflammation in RA patients treated with tofacitinib, suggesting the potential utility of ultrasonography for disease monitoring. Future larger-scale RA studies using a prospective longitudinal study design, with clinical and imaging assessments performed at multiple time-points, will be required to evaluate the performance (including the sensitivity to change) of these patient-level ultrasound joint inflammation outcomes (derived from the EULAR-OMERACT scoring system) as monitoring tools in RA patients with different clinical/treatment profiles, such as those with early versus long-standing disease or those on conventional versus biological DMARD therapy. In the present study, we tested the construct validity of patient-level ultrasound scores based on the EULAR-OMERACT system by comparing them with clinical indices and structural damage. Although not examined in our present study, these patient-level ultrasound scores based on the EULAR-OMERACT system could also be compared to other imaging modalities (such as MRI, which detects both joint inflammation and damage) and non-imaging outcome measures (such as novel biomarkers and functional status assessments).

In conclusion, we have added to the RA imaging literature by demonstrating that patient-level ultrasound joint inflammation outcomes derived from the EULAR-OMERACT joint-level scoring system exhibit good construct validity when compared with both disease activity and joint damage in patients with RA. Our next step is to explore their responsiveness as outcome measures in longitudinal RA study cohorts.

## Data Availability

The datasets presented in this article are not readily available because of confidentiality. Requests to access the datasets should be directed to York Kiat Tan, tan.york.kiat@singhealth.com.sg.
